# Choosing a career in oncology: results of a nationwide cross-sectional study

**DOI:** 10.1186/s12909-018-1117-2

**Published:** 2018-01-15

**Authors:** J. C. Faivre, J. E. Bibault, A. Bellesoeur, J. Salleron, M. Wack, J. Biau, M. Cervellera, G. Janoray, T. Leroy, N. Lescut, V. Martin, S. Molina, B. Pichon, C. Teyssier, S. Thureau, J. J. Mazeron, H. Roché, S. Culine

**Affiliations:** 1 Academic Radiation Oncology & Brachytherapy Department, Lorraine Institute of Cancerology - Alexis-Vautrin Comprehensive Cancer Centre, 6 avenue de Bourgogne, 54519 Vandœuvre-lès-Nancy, France; 2 French Society of Young Radiation Oncologists (SFjRO), Centre Antoine-Béclère, 45 rue des Saint Pères, 75005 Paris, France; 3 Radiation Oncology Department, University Hospital of Paris (Georges Pompidou European Hospital), 20 rue Leblanc, 75015 Paris, France; 4 Paris Descartes University, 12 rue de l’Ecole de médicine, 75006 Paris, France; 5 Medical Oncology Department, University Hospital of Paris (Teaching Hospital Cochin), 27 rue du Faubourg Saint Jacques, 75014 Paris, France; 6 French Resident’s and Fellow’s Association for Teaching and Research in Oncology (AERIO), 149 avenue du Maine, 75014 Paris, France; 7 Biostatistics Department, Lorraine Institute of Cancerology - Alexis-Vautrin Comprehensive Cancer Centre, 6 avenue de Bourgogne, F-54519 Vandœuvre-lès-Nancy, France; 8 Biostatistics and Epidemiology Department, University Hospital of Nancy, 9 avenue de la Forêt de Haye, 54505 Vandoeuvre-lès-nancy, France; 9 Radiation Oncology Department, Jean-Perrin Comprehensive Cancer Centre, 58 rue Montalembert, 63000 Clermont-Ferrand, France; 10 University of Auvergne, 28 place Henri Dunant, 63000 Clermont-Ferrand, France; 11 Radiation Oncology Department, Jean Godinot Comprehensive Cancer Centre, 1 rue du Général Koenig, 51726 Reims, France; 12 S. Kaplan Cancer Centre, Radiation Oncology Department, University Hospital of Tours, 2 boulevard Tonnelé, 37000 Tours, France; 13 Academic Radiation Oncology Department, Oscar Lambret Comprehensive Cancer Centre, 3 rue Frédéric Combemale, 59000 Lille, France; 14 Radiation Oncology Department, University Hospital of Besançon, 3 boulevard Fleming, 25000 Besançon, France; 15 Radiation Oncology Department, University Hospital of Paris (Kremlin-Bicêtre Hospital), 78 rue du Général Leclerc, 94270 Paris, France; 16 Paris Sud University, 63 rue Gabriel Péri, 94276 Orsay, France; 17 Radiation Oncology Department, University Hospital of Poitiers, 2 rue de la Milétrie, 86021 Poitiers, France; 18 Radiation Oncology Department, René-Gauducheau Comprehensive Cancer Centre, boulevard Jacques Monod, 44805 Nantes, Saint-Herblain France; 19 Radiation Oncology & Medical Physics Department, Henri-Becquerel Comprehensive Cancer Centre, rue d’Amiens, 76000 Rouen, France; 20 EA4108 QuantIf Litis, University of Rouen, 22 boulevard Gambetta, 76000 Rouen, France; 21 Radiation Oncology Department, University Hospital of Paris (Pitié-Salpétrière Hospital), 83 boulevard de l’hôpital, 75013 Paris, France; 22 University Pierre et Marie Curie, 4 place Jussieu, 75005 Paris, France; 23 Oncopole Toulouse, Claudius Regaud Comprehensive Cancer Centre, 1 avenue Irène Joliot-Curie, 31059 Toulouse, France; 24 University of Toulouse, 37 allée Jules Guesde, 36000 Toulouse, France; 25 Medical Oncology Department, University Hospital of Paris (Saint-Louis Hospital), 1 avenue Claude Vellefaux, 75010 Paris, France; 26 Paris Diderot University, 16 rue Huchard, 75018 Paris, France; 27 Academic Department of Radiation Therapy & Brachytherapy, Lorraine Institute of Cancerology – Alexis-Vautrin CLCC [Centre de lutte contre le cancer – Cancer Centre] – Unicancer, 6 avenue de Bourgogne –CS 30 519, cedex F-54 511 Vandoeuvre-lès-Nancy, France

**Keywords:** Internship and residency, Education, Training, Research, Career mobility, Neoplasms

## Abstract

**Background:**

Little information is currently available concerning young medical students desire to pursue a career in oncology, or their career expectations.

**Methods:**

This project is a cross-sectional epidemiological study. A voluntary and anonymous questionnaire was distributed to all young oncologists studying in France between the 2nd of October 2013 and the 23rd of February 2014.

**Results:**

The overall response rate was 75.6%. A total of 505 young oncologists completed the questionnaire. The main determining factors in the decision to practice oncology were the cross-sectional nature of the field (70.8%), the depth and variety of human relations (56.3%) and the multi-disciplinary field of work (50.2%). Most residents would like to complete a rotation outside of their assigned region (59.2%) or abroad (70.2%) in order to acquire additional expertise (67.7%). In addition, most interns would like to undertake a fellowship involving care, teaching and research in order to hone their skills (85.7%) and forge a career in public hospitals (46.4%). Career prospects mainly involve salaried positions in public hospitals. Many young oncologists are concerned about their professional future, due to the shortage of openings (40.8%), the workload (52.8%) and the lack of work-life balance (33.4%).

**Conclusions:**

This investigation provides a comprehensive profile of the reasons young oncologists chose to pursue a career in oncology, and their career prospects.

**Electronic supplementary material:**

The online version of this article (10.1186/s12909-018-1117-2) contains supplementary material, which is available to authorized users.

## Background

**Fig. 1:**
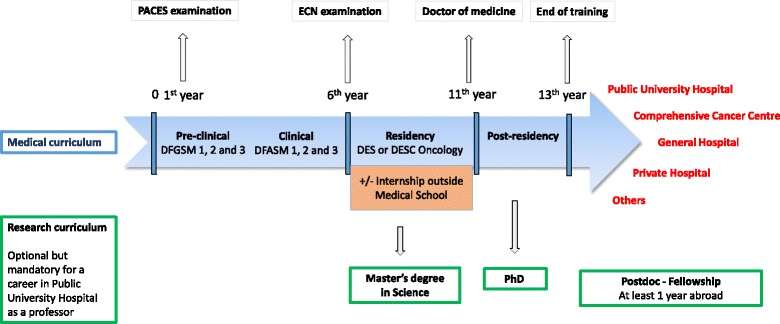
Medical and research curricula in France. Footnotes: PACES: [Première Année Commune des Etudes de Santé]: First Common Year for Health Studies. An end-of-year examination obligatory for the continuation of medical studies. DFGSM and DFASM: [Diplômes de Formations Générales ou Approfondies en Sciences Médicales]: Diplomas of General or Advanced Training in Medical Sciences. ECN: [Epreuves Classantes Nationales]. Students can enrol in national ranking exams (called ECN) and choose according to their ranking position a specialty and an assigned health region in which to complete their internship. Residency: Access to a career in oncology in France is possible with two types of diploma: the DES for a duration of 5 years with three options: radiation oncology, medical oncology or onco-haematology; and the DESC. The DES diploma qualifies practitioners and confers exclusive practice. The DESC in oncology is open to holders of the various DESs in medical or surgical specialities, certifies specific additional training in oncology, and confers limited practice of cancerology in the field of the initial DES (pulmonology, hepato-gastroenterology, etc.). Post-residency: Residents usually complete their training with a post-residency position to hone their skills and develop professional autonomy

The annual restriction on the number of doctors in training means that access to medical studies is limited. The study of medicine in France is arranged over three periods presented in Fig. 1: a first (pre-clinical) period of 3 years, a second (clinical) period of 3 years, and a third period (residency) of 4 to 5 years, specific to each specialty. At the end of the second period, students can enrol in national ranking exams, called Epreuves Classantes Nationales (ECN), and according to their ranking position choose a specialty and a medical school (health region assigned for the entire residency) in which to complete their internship. However, after acceptance on a training program and its funding, residents can spend one internship outside their medical school or abroad to acquire specific expertise and complete their oncology training. Yet the number of accepted and financed external internships may still be too low to accommodate all those residents wanting to apply. Residents usually complete their training with a post-residency position to hone their skills and develop professional autonomy. In addition to the regular medical curriculum, during their residency young oncologists can also undertake an additional period of scientific training that can be used to gain a Master’s Degree, a PhD and/or a year abroad.

Little information is currently available concerning young medical students desire to pursue a career in oncology. In 2011, Loriot et al. [1] published a study that included only a small population of young medical oncologists. The strongest factors that had influenced their decision to become a medical oncology specialist were an interest in medical oncology, exposure to this branch of medicine during graduate training as a medical student, an interest in research, and the diverse subject area. The most popular prospective career choice was working in a public hospital, but most residents stated that they had not received adequate information regarding the different potential career paths open to them. We intended the scope of our study to be wider than that of previous studies, and we included medical, radiation, and haematology oncologists and organ specialists. No data are currently available regarding working hours (i.e. full-time or part-time) and young oncologists’ concerns about their professional future. Other subjects that have not previously been studied regarding the career development of young oncologists include: internships outside of their medical school during residency in France and/or abroad, post-residency positions, or the role of scientific societies in professional development. Moreover, data are not available concerning career expectations or determining factors in the decision to practice oncology, radiation oncology, onco-haematology or organ-oriented specialities in the treatment of cancer patients. Recently, we studied theoretical training in more detail [2]. In this study we showed that residents needed additional training, due to the shortage of specialized postgraduate degree training. Existing teaching topics that were deemed to be in need of improvement were: basic concept, advanced concept and the discussion of frequent clinical cases. The topics not covered that needed to be taught were: career development, medical English, organization of the radiation oncology (RO) speciality and hospital management of the RO department.

The nationwide investigation presented in the current study had several objectives:

- Describe the determining factors in the decision to practice oncology.
- Assess the appeal to young oncologists of a residency outside their assigned region in France and/or abroad.
- Describe the career expectations of young oncologists: post-residency, research and first position.

## Methods

### Participants

We included all young oncologists in France enrolled in programs leading to the Diplôme d’Etudes Spécialisées [Diploma of Specialized Studies] (DES) in Oncology and the Diplôme d’Etudes Spécialisées Complémentaires [Diploma of Complementary Specialized Studies] (DESC) in Cancerology with a valid email address as of the 1st of May 2013, regardless of internship, semester or university.

#### Survey

This project is a French nationwide cross-sectional epidemiological study based on exhaustive sampling.

The questionnaire was only available online (additional file 1) and was used to collect:

- The sociodemographic profile of young oncologists: age, gender, marital status, name of medical school and speciality.
- Determining factors in the decision to practice oncology.
- The appeal of and reasons for pursuing an internship outside of their assigned health region in France and/or abroad.
- Career expectations: post-internship (status), and first position (status, working hours and concerns about their future career).
- Research (clinical, translational, fundamental): validation of Master’s degree, discipline, funding, scientific publication, poster and/or oral presentation.
- Role of national and international learned societies in professional development.

The questionnaire consisted of 50 questions: 25 single choice questions, 19 multiple choice questions, 5 visual analogue scales and 1 free comments section.

### Implementation

The questionnaire was a standardized anonymous electronic questionnaire designed by a young radiation oncologist from the Société Française des jeunes Radiothérapeutes Oncologues (French Society of young Radiation Oncologists, SFjRO) alongside a statistician. A working group piloted the questionnaire. The questionnaire was distributed using Google Docs between the 2nd of October 2013 and the 23rd of February 2014, followed by three reminders, using the national intern network’s email list.

### Statistical analysis

All of the data collected were analysed (both complete and incomplete responses). A comparison between the number of respondents and the number of residents by speciality and by faculty was performed based on the number of residents by faculty and speciality according to the National Office of Health Profession Demographics, in order to ensure that respondents were representative of the source population.

All of the analyses were conducted using R-3.1.1, with a 5% risk of type I errors. Qualitative variables were described using frequency and percentage. Comparisons were performed using Chi-square tests.

### Ethics

The questionnaire was voluntary. The study did not collect any identifying information (e.g., name, address, email address, etc.) so that the information can never be linked to the respondents who supplied it, thus ensuring the anonymity of the respondents. This study was declared to the French Data Protection Authority (“Commission Nationale de l’Informatique et des Libertés”).

### Funding

Authors or participants received no funding for this study, but the article-processing charge was supported by the SFjRO.

## Results

### Population

The baseline characteristics of the respondents are presented in Table 1. A total of 505 young oncologists responded to the online survey, which meant an overall response rate of 75.6% (505/668). Specifically, the response rates were 72.7% (304/418) for the DES in Oncology and 80.4% (201/250) for the DESC in Oncology.

**Table 1:** Baseline characteristics of the respondents

Characteristics	% (n/N)
Male	47.8 (241/504)
Status	
Intern	80.4 (406/505)
Assistant Professor	16.0 (81/505)
Hospital Assistant Specialist	3.6 (18/505)
Specialities	
DES in Oncology	60.2 (304/505)
medical oncology option	20.8 (105/505)
radiation oncology option	36.0 (182/505)
haematology/oncology option	3.4 (17/505)
Other DES in the DESC in Cancerology	39.8 (201/505)
Family situation	
Couple	70.3 (353/502)
Spouse’s socio-professional category	
Doctor	47.2 (167/354)
Manager, professional	28.8 (102/354)
Intermediate professional	9.0 (32/354)
Craftsperson, merchant, business manager	1.4 (5/354)
Employee	5.9 (21/354)
Labourer	0.0 (0/354)
Student	6.2 (22/354)
Looking for employment	1.4 (5/354)
Child(ren)	20.5 (103/503)

### Determining factors in the decision to practice oncology

The main determining factors in the decision to practice oncology are presented in Table 2 and were the cross-sectional nature, the depth and variety of human relations, the multi-profession / multi-disciplinary field of work and the clinical aspect of the work. The depth and variety of human relations refers to the set of interactions between caregivers, patients and their families. They are based on the links which exist between people, and which occur through both verbal and non-verbal communication. The cross-sectional nature of oncology refers to the wide range of knowledge/skills/sub-specialities required to practice oncology.

**Table 2:** Determining factors in the decision to practice oncology

	Total % (n) *N* = 504	DES Oncology % (n) *N* = 304	Radiation oncology % (n) *N* = 182	Onco-haematology % (n) *N* = 17	Medical oncology % (n) *N* = 105	Other DES % (n) *N* = 200	Overall p
Cross-sectional nature	70.8 (357)	74.7 (227)	73.1 (133)	64.7 (11)	62.9 (66)	35.5 (71)	0.05
Depth and variety of human relations	56.3 (284)	65.8 (200)	57.1 (104)	76.5 (13)	79.0 (83)	42.0 (82)	*p* < 0.001
Multi-profession and multi-disciplinary field of work	50.2 (253)	59.9 (182)	57.7 (105)	64.7 (11)	62.9 (66)	35.5 (71)	*p* < 0.001
Clinical	49.6 (250)	44.1 (134)	38.5 (70)	52.9 (9)	52.4 (55)	58.0 (116)	*p* < 0.001
Technical level	44.0 (222)	46.4 (141)	67.0 (122)	11. 8 (2)	16.2 (17)	40.5 (81)	*p* < 0.001
ECN ranking	41.9 (211)	44.1 (134)	50.0 (91)	23.5 (4)	37.1 (39)	38.5 (77)	0.03
Research	35.9 (181)	37.8 (115)	26.9 (49)	58.8 (10)	53.3 (56)	33.0 (66)	*p* < 0.001
Work-life balance	35.7 (180)	50.7 (154)	65.9 (120)	5.9 (1)	31.4 (33)	13.0 (26)	*p* < 0.001
Geographic ties	20.6 (104)	18.1 (55)	18.7 (34)	17.6 (3)	17.1 (18)	24.5 (49)	0.39
Personalized medicine	19.2 (97)	20.4 (62)	12.6 (23)	35.3 (6)	31.4 (33)	17.5 (35)	*p* < 0.001
Family ties	19.0 (96)	16.1 (49)	15.9 (29)	17.6 (3)	16.2 (17)	23.5 (47)	0.29
Working conditions	17.7 (89)	23.4 (71)	34.1 (62)	5.9 (1)	7.6 (8)	9.0 (18)	*p* < 0.001
Remuneration	12.5 (63)	19.1 (58)	26.9 (49)	0 (0)	8.6 (9)	2.5 (5)	*p* < 0.001

ECN (Epreuves Classantes Nationales). Overall p = overall comparisons between the four groups (Radiation oncology, Onco-haematology, Medical oncology and Other DES)

### Internship outside their assigned region in France and/or abroad

Respectively, 59.2% (299/505) and 70.2% (354/505) of young oncologists would like to spend six months or a year either outside of their assigned health region in France or abroad. Their main motives were to learn other practices in different services (81.6% (283/347)) and to acquire expertise not available in their original region (67.7% (235/347)). Other reasons were poorly represented (to obtain a post-residency position, to develop a research project between the two hospitals, family or geographic ties).

### Post-residency

Of the oncology interns questioned 89.3% (453/505) would like to continue their training with a hospital fellowship, 10.3% (52/505) were undecided, and 0.4% (2/505) were not interested in pursuing a hospital fellowship. The most desired type of fellowship status was that of Assistant Professor involving care, teaching and research (69.7% (352/505)). The next most desired post was Hospital Assistant Specialist involving care only (17.3% (86/505)). The main reasons given for undertaking a fellowship were: complete/further education (85.7% (382/505)); desire for a career in a public hospital or cancer centre (46.4% (207/505)); desire for a career in a university hospital (39.9% (178/505)); to validate a DESC in Oncology (38.8% (173/505)); and desire for a career in private practice (28.5% (127/505)).

### Career expectations

The career expectations of the participants are presented in Table 3.

**Table 3:** Career expectations of the respondents

Career expectations	% (n/N)
Career in a public university hospital centre or a cancer centre that does not involve teaching or research	48.3 (244/505)
Career as a doctor/professor	39.0 (197/505)
Career in a general hospital	35.0 (177/505)
Career in a private hospital	34.3 (173/505)
Career in the health care industry	2.4 (12/505)

More men than women were interested in a career as doctor/professor that involves teaching and research in a public university hospital or a cancer centre (48.5% (117/241) of men versus 30.4% (80/263) of women, *p < 0.001*), or in a career in a private hospital centre (41.5% (100/241) of men versus 27.8% (73/263) of women, *p = 0.002*), or in the pharmaceutical industry (2.9% (7/301) of men versus 0.4% (1/263) of women, *p = 0.031*).

Among young oncologists, 72.6% (366/504) would like to work full-time, 25.2% (127/504) would like to work in a part-time position (between 70% and 90% full-time equivalent) and 2.2% (8/504) would like to work in a half-time position. More women than men want to work part-time (38.8% (102/263) versus 13.7% (73/263), *p < 0.001*).

The young oncologists’ main concerns for the future are presented in Table 4.

**Table 4:** The respondents concerns for the future

	Total % (n) *N* = 449	DES Oncology % (n)0 *N* = 270	Radiation oncology % (n) *N* = 160	Onco-haematology % (n)0 N = 17	Medical oncology % (n) *N* = 93	Others DES % (n) *N* = 179	Overall p
Workload too heavy	52.8 (237)	50.4 (136)	46.2 (74)	52.9 (9)	57.0 (53)	56.4 (101)	0.23
Shortage of openings	40.8 (183)	42.2 (114)	41.2 (66)	52.9 (9)	41.9 (39)	38.5 (69)	0.69
Lack of work-life balance	33.4 (150)	27.4 (74)	26.2 (42)	58.8 (10)	23.7 (22)	42.5 (76)	p < 0.001
Insufficient income	24.5 (110)	22.6 (61)	17.5 (28)	17.6 (3)	32.3 (30)	27.4 (49)	0.04
Not what I want to do	24.1 (108)	25.6 (69)	25.0 (40)	17.6 (3)	28.0 (26)	21.8 (39)	0.66
Uncertainty surrounding freedom of establishment	20.5 (92)	22.2 (60)	28.8 (46)	0 (0)	15.1 (14)	17.9 (32)	p < 0.001
Shortage of openings for spouse	16.9 (76)	18.1 (49)	20.6 (33)	11.8 (2)	15.1 (14)	15.1 (27)	0.51
Professional isolation	6.9 (31)	9.6 (26)	8.1 (13)	11.8 (2)	11.8 (11)	2.8 (5)	0.01

Overall p = overall comparisons between the four groups (Radiation oncology, Onco-haematology, Medical oncology and Other DES)

### Research (clinical, translational, fundamental)

Some 33.1% (167/505) of young oncologists had dedicated or were currently devoting a year to research (earning them a Master’s degree in addition to their medical curriculum). Of these, 52.7% (76/146) and 55.5% (81/146) would like to follow up their research with a PhD and/or a year abroad, respectively. The reasons for dedicating a year to medical research are presented in Table 5. Some 32.7% (52/505) of young oncologists who had dedicated or who were currently devoting a year to medical research did not receive funding.

**Table 5:** Determining factors in the decision to complete a Master’s degree in addition to the medical curriculum

	Total % (n) *N* = 166	DES Oncology % (n) *N* = 94	Radiation oncology % (n) *N* = 45	Onco-haematology % (n) N = 4	Medical oncology % (n) *N* = 45	Others DES % (n) *N* = 72	Overall p
Openness, curiosity, interest in research	71.7 (119)	70.2 (66)	64.4 (29)	50 (2)	77.8 (35)	73.6 (53)	0.33
Training complementary to research	50.0 (83)	41.5 (39)	31.1 (14)	25.0 (1)	53.3 (24)	61.1 (44)	0.008
Career in a university hospital	47.0 (78)	45.7 (43)	57.8 (26)	50.0 (2)	33.3 (15)	48.6 (35)	0.12
Prerequisite for fellowship	42.8 (71)	37.2 (35)	33.3 (15)	75.0 (3)	37.8 (17)	50.0 (36)	0.14
Expectation of a fellowship	9.0 (15)	9.6 (9)	13.3 (6)	0 (0)	6.7 (3)	8.3 (6)	0.73
Obligation for diploma	7.8 (13)	9.6 (9)	8.9 (4)	0 (0)	11.1 (5)	5.6 (4)	0.64

Overall p = overall comparisons between the four groups (Radiation oncology, Onco-haematology, Medical oncology and other DES)

Of those young oncologists that had already dedicated a year to medical research, a minority of them indicated that they wanted a career in a private hospital centre (18.6% (31/167) versus 43.5% (77/177) who did not, *p < 0.001*) or a career in both the public and private health care sectors (26.9% (45/167) versus 45.8% (81/177) who did not, *p < 0.001*). Most of the young oncologists that had already dedicated a year to medical research indicated that they wanted a career as a doctor/professor, regardless of whether the position involved teaching (56.3% (94/167) versus 38.4% (68/177) who did not, *p = 0.003*) or research (61.7% (103/167) versus 11.9% (21/177) who did not, *p < 0.001*).

Research studies were published in 19.6% (31/158), and/or presented orally in 26.6% (42/159), or in poster form in 38% (60/158) of cases. Marital status did not affect career expectations (*p* > 0.05).

### Scientific societies

In all, 32.2% (148/459) of young oncologists declared they belonged to a scientific society. The main learned societies to which young oncologists belong are the French Society of Radiation Oncologists (SFRO) (8.1% (37/459)) and the European Society for Therapeutic Radiology and Oncology (ESTRO) (9.8% (45/459)).

## Discussion

### Main findings

Our research is a large comprehensive questionnaire study investigating the motivation of young oncologists to pursue a career in oncology and their career concerns and expectations, with a particular interest in reasons for pursuing an academic career. The main determining factors in the decision to practice oncology are the cross-sectional nature, the depth and variety of human relations and the multi-disciplinary field of work. Most residents would like to undertake a rotation outside their assigned health region or abroad, in order to acquire additional expertise. In addition, most interns would like to take a fellowship as an Assistant Professor involving care, teaching and research in order to hone their skills and forge a career in public hospitals, for the most part full-time. More than a third of young oncologists are interested in research (clinical, translational, fundamental). Career prospects mainly involve salaried positions in public hospitals. The future of oncology is a source of concern for a large number of young oncologists due to the shortage of openings, the heavy workload and the lack of work-life balance.

### Strengths and limitations

The strengths of our study are that we included a larger number of respondents than has previously been explored (*n* = 505, 75.6% overall response rate), and in addition our study assessed a wide range of themes. Indeed, we studied some subjects in more detail than the study by Loriot et al. [3] including: sociodemographic profiles, determining factors in the decision to practice oncology, work expectations (working hours and concerns about professional future). Other subjects have never before been studied for young oncologists: internship during their residency either outside of their medical school in France or abroad, post-residency positions, and the role of teaching societies in professional development. A further strength of our study is that a wide range of young oncologists participated, thus allowing a comparison between medical oncologists, radiation oncologists, haematologists and organ specialists. One limitation of our study is that the results are not comparable with other countries because the training of doctors is partially different from one country to another.

### Determining factors in the decision to practice oncology

The main determining factors in the decision to practice oncology were the cross-sectional nature, the depth and variety of human relations and the multi-profession / multi-disciplinary field of work. These determinants can be considered as the “foundations” of oncology. Epreuves Classantes Nationales ranking, family and geographical ties were not determining factors in the decision to practice oncology. In particular, young radiation oncologists are attracted by the technical aspect of radiation oncology.

G. Coindard revealed the importance of the physician-patient relationship and clinical research in choosing a career in medical oncology [4]. Loriot et al. identified four determining factors in the decision to practice medical oncology: an interest in oncology, completing a rotation in an oncology department during the first two years of medical training, research opportunities, and variety in the clinical practice [1]. Earlier, Kantor et al. identified these factors, as well as the technical aspect involved in a career as a radiation oncologist [5]. Internship in an oncology department in the first two years of medical training was not identified in the earlier study. Aneja et al. identified the role of ranking medical students in the United States by the National Residency Matching Program (NRMP), the equivalent of the Examen National Classant [National Ranking Test] in France, to enter a residency program in radiation oncology [6]. Internships in radiation oncology are competitive and selective. To our knowledge, there is no easily accessible data for the other oncology specialities. Mattes et al. found that the main factors considered by young oncologists when they apply for a position are the possibility of collegial discussion, geographic location, the quality of patient care, organizational and logistical aspects, and the multi-disciplinary approach [7].

### Internship outside their assigned region in France and/or abroad

Most young oncologists would like to spend six months or a year outside their medical school (assigned health region for the entire residency) or abroad. Currently, the number of internships accepted and financed is still probably too low to accommodate all the prospective residents and training projects, hence more resources should be dedicated. As expected, learning other practices and expertise not available in their medical school were the major incentives to undertake an internship outside their assigned medical school, most often in a renowned oncology department. Whilst obtaining a post-residency position was not a major determining factor in the decision to complete an internship outside their assigned region, it is a way to make oneself acquainted with potential recruiters. In the same way, family or geographical ties were also not major determining factors, although this would seem natural.

### Post-residency

Most students would like to follow their internship up with a fellowship as an Assistant Professor to complete their training and obtain a position in a university hospital. This was also identified by Dewas et al. in 2009 [8]. The fellowship enables students to further their training, narrow their speciality, earn a higher salary and/or obtain an academic position. J. Leung et al. identified that most trainees are interested in fellowship positions, links with academia and largely public sector work in the future. However, job availability in the future is a major concern [9]. Zaorsky et al. highlighted the responsibilities of chief residents in the United States, involving care, teaching, research and management [10]. These findings show that young oncologists, even after many years of specialized training, are still interested in furthering their education. We believe that this is due to the ceaseless ongoing changes in the knowledge and techniques surrounding oncology [11].

### Career expectations

Excessive workload and shortage of post-residency positions are major concerns, whatever the speciality. The first is an important factor, especially considering our investigation published in 2010 emphasizing the prevalence of burnout in young oncologists [12]. Indeed, work-life balance is a major concern especially for onco-haematologists, as a very high workload is often observed in onco-haematology departments.

More women than men want to work part-time, probably because women often need more personal time due to family responsibilities. In addition, more men than women are interested in a career as a doctor/professor in a public university hospital. According to Chang et al., the determining factors in choosing an academic career are interest in an academic career before internship, the image reflected by academics during their medical studies and opportunities to work in research during their internship [13]. The interest of young interns in pursuing an academic career diminished over time. In a literature review conducted by Borges et al., the main factors physicians consider when choosing an academic career are personal values, financial considerations (the cost of education and remuneration), gender, the image reflected by academics and interest in research [14]. Additional factors associated with choosing an academic career included factors related to mentorship, intellect, and field of research.

Fellows selecting non-academic careers prioritized lifestyle in their career decisions [15]. However, according to Chang et al. the factors associated with the choice of a career in the private health care sector are: interest in a career in the private health care sector, the image reflected by academics, and academic requirements and pressures [13]. Lifestyle, the quality of the working environment, the quality of patient care, geographic location and enjoyable working relationships with colleagues are determining factors in the choice of a career in the private health care sector [13].

### Research (clinical, translational, fundamental)

Most young oncologists are interested in research. Nevertheless, research and appropriate mentoring are important factors for young oncologists interested in an academic career. L. Horn et al. suggest fellows choosing an academic career were more likely to have presented and published their research [15]. Wilson et al. suggest that participating in academic activities during an internship was associated with the choice of an academic career [16]. However, many young oncologists decide not to undertake a research project due to a lack of funding.

### Scientific societies

Few young oncologists belong to international teaching societies, particularly in medical oncology. This could be explained by the membership fees, the language barrier or the lack of sponsorship. More ambitious and original policies specifically aimed at young oncologists could promote their attraction to and involvement in learned societies.

### Prospects

This investigation provides the first ever nationwide profile of the reasons for practicing oncology, as well as the career paths and career prospects of young oncologists. The implications for medical practice could include:

- Fostering biomedical research training during internship, which is considered appealing for a career in hospitals.
- Fostering access to internships outside of their assigned health region in France and/or abroad in order to diversify the professional experience acquired during an internship, and open up opportunities for international study for young oncologists.
- Fostering access to fellowships before the first career position in order to further training.
- Promote membership to academic oncology societies among residents.

## Conclusion

The main determining factors in the decision to practice oncology are the cross-sectional nature of the field, the depth and variety of human relations and the multi-disciplinary field of work. Most residents would like to spend six months or a year outside their assigned health region or abroad in order to acquire additional expertise. In addition, most residents would like to have a fellowship as an assistant professor involving care, teaching and research in order to hone their skills and forge a career in public hospitals, for the most part full-time. More than a third of young oncologists are interested in research.

## Additional file

Additional file 1: Questionnaire of the survey. (DOCX 52 kb)
