# Supplementary material for: Choosing a career in oncology: results of a nationwide cross-sectional study
Source: BMC Med Educ. 2018 Jan 15;18:15. doi: 10.1186/s12909-018-1117-2 (PMC5769332; doi:10.1186/s12909-018-1117-2)
Supplement: Additional file 1: — Questionnaire of the survey. (DOCX 52 kb) [file 12909_2018_1117_MOESM1_ESM.docx]

**Additional data: questionnaire of the survey**

**French national survey of young oncologists**

Thank you for taking the time to answer the French national survey of young oncologists

Thank you for your involvement !

**PERSONAL PROFILE**
1) How old are you? (only one answer)

23

24

25

26

27

28

29

30

31

32

33

34

35 and more

2) What is your gender? (only one answer)
 man
 woman

3) Which is your marital situation? (only one answer)
 single person
 in couple

4) Do you have children? (only one answer)
Conditional: If yes, go to question 5, if not, go to question 6.
 yes
 no

5) How many children do you have? (only one answer)
 1
 2
 3 and more

6) If you don’t have child, is it because of your profession? (Only one answer)
 yes
 no

7) What is your status? (mandatory question, one answer)
 resident

resident with supplemental year called “Gold Medal”)

assistant professor

hospital assistant specialist

other

8) What is your medical school?
(mandatory question, only one answer)

Amiens

Angers

Antilles-Guyane

Besançon

Bordeaux

Brest

Caen

Clermont-Ferrand

Dijon

Grenoble

Lille

Limoges

Lyon

Marseille

Montpellier – Nîmes

Nancy

Nantes

Nice

Océan Indien

Paris

Poitiers

Reims

Rennes

Rouen

Saint-Etienne

Strasbourg

Toulouse

Tours

9) In wich speciality are you enrolled?
 Medical Oncology (conditional skip to Question 11)
 Radiation Oncology (conditional skip to Question 11)
 Onco-hematology (conditional skip to Question 11)
 another specialty (Conditional: go to question 10)

10) What is your speciality?

pathologists
 anesthesia - resuscitation
 medical biology
 heart and vascular diseases
 plastic surgery, cosmetic and reconstructive
 pediatric surgery
 maxillofacial surgery
 oral surgery
 orthopaedic surgery and traumatology
 thoracic and cardiovascular surgery
 urology
 vascular surgery
 digestive surgery
 dermatology
 endocrinology, diabetology and metabolic diseases
 genetics
 medical gynecology
 obstetric gynecology
 hematology
 hépatogastro-enterology
 occupational medicine
 internal medicine
 nuclear medicine
 physical medicine and rehabilitation
 nephrology
 neurosurgery
 neurology
 ophthalmology
 otorhinolaryngology
 pediatrics
 pneumology
 psychiatry
 radiology and medical imaging
 rheumatology
 public health

11) What is your DESC (Specialized Studies Diploma Supplement) and if so which one?
 DESC of Oncology (conditional skip to Question 12)
 DESC of pain medicine and palliative care (conditional skip to Question 13)
 not registered in a DESC (conditional skip to Question 13)
 other:

12) What is your overspecialization of DESC of Oncology?

medical treatment of cancers
 cancer surgery
 supportive care
 cancer imaging
 biology of cancers

13) What were the determining factors on the decision to practice oncology? (Multiple answers possible)

the ranking ECN
 geographical ties
 family ties
 remuneration
 quality of life / work-family balance
 working conditions
 richness of human relations
 technical level
 clinical
 research
 cross-sectional nature
 multidisciplinary and multiprofession nature
 personalized medicine: cellular and molecular biology, genetics
 other:

14) Did you changed of your initial faculty of medicine for residency?
 yes (conditional skip to Question 15)
 not (conditional skip to Question 16)

15) If yes, why did you change?
 to adapt to my result at EXN ranking
 for better training
 for personal reasons
 other:

**RESIDENCY: INTERNSHIP IN PRIVATE HOSPITAL AND OUTSIDE ASSIGNED REGION IN FRANCE OR STAGE ABROAD**

16) Why do you think that it is interesting to do an internship in the private hospital? (Multiple answers possible)
 cope with the increasing numerus clausus and saturation of internship
 discover the private practice
 support other pathologies encountered little or no to the public hospital
 create a public - private emulation for the training of the interns
 no
 without opinion
 other:

17) Which risk do you see in open internships in private hospital? (Multiple answers possible)
 replacing cheap / decrease replacements opportunities
 no respect for the dedicated time for training
 no respect of safety rest
 lack of accountability
 supervisory failure
 insufficient research
 patient refusal to be seen by an internal
 presence of non-assumed internal to the patient base
 no guards and penalties
 no
 without opinion
 other:

18) Are you interested in an internship in private hospital?
0 1 2 3 4 5 6 7 8 9 10
select a value from 0 = not at all interested to 10 = very interested

19) For you, a half-year of training in the private hospital during the residency should be (only one answer) :
 mandatory in the
 highly recommended
 optional
 no place
 I do not know

20) Have you ever done or would you make replacements? (Only one answer)
 yes
 no
 i do not know

21) For which reason you did you replaced?
(Multiple answers possible)
 additional training
 income supplement
 participation to the night duty
 to discover private healthcare hospital
 prepare installation in private - to build a network
 gain autonomy
 finance your theoretical training
 other:

22) Have you ever done or do you want to do an intership outside my assigned health region in France? (Only one answer)
 yes
 no
 I do not know

23) Why did you or would you make an internship outside your assigned health region in France? (Multiple answers possible)
 insufficient training courses in my health region in France
 in order to obtain a post-residency position
 acquire skills not available in my health region in France
 learn practices from different department of oncology
 develop a research project between the two hospitals
 family or geographical ties
 other:

24) Would you exercise a half-year abroad if possible? (Only one answer)
 yes
 no

**LEARNED SOCIETIES and ASSOCIATIONS OF YOUNG DOCTORS**

25) Are you member of a learned society? (Multiple answers possible)
 no

SFC

SFRO

ESMO

ESTRO

EACR

ASCO

ASTRO

AACR

other:

26) Are you a member of a national association of residents? (Multiples answers are possible)
 no

SFjRO

Collège National des Internes d'Oncologie Médicale

AJCO

AFJDSP

Anatomopathologie - AFIAP

Anesthésie - Réanimation - ANICAR

Cardiologie - GCF

Chirurgie infantile - ACPF

Chirurgie maxillo-faciale - AJCMF

Chirurgie orale - ANICO

Chirurgie orthopédique - CJO

Chirurgie thoracique et cardio-vasculaire - JCTCV

Chirurgie urologique - AFUF

Chirurgie vasculaire - SICCV

Chirurgie viscérale - AJCV

Dermatologie - AJDerm

Endocrinologie - Diabétologie - UNITED

Génétique médicale - SIGF

Gynécologie médicale - AIGM

Gynécologie Obstétrique - AGOF

Hématologie - AIH

HGE - AFIHGE

Médecine interne - Amicale des jeunes internistes

Médecine nucléaire - ANAIMEN

Médecine Physique Rééducation - AJMER

Néphrologie - SNN

Neurochirurgie

Neurologie - ANAINF

Ophtalmologie - ANJO

Pédiatrie - AJP

Pneumologie - AJPO2

Psychiatrie - AFFEP

Radiologie - UNIR

Rhumatologie - ARF

Santé publique et médecine sociale - CLISP

other:

27) Are you member of a regional association of residents? (Multiple answers possible)
 no

AERIO (Paris -Ile de France)

AFICOA (Clermont-Ferrand

AJOI (Grenoble)

AJOM (Marseille)

AJON (Nice)

AJORA (Lyon - Saint-Etienne)

ANEICCOH (Strasbourg, Nancy, Besançon, Reims, Dijon)

ANICOR (Lille)

ANOORM (Rennes, Poitiers, Brest, Angers, Tours, Poitiers)

ASIO (Toulouse, Bordeaux, Limoges, Montpellier)

other:

**POST-RESIDENCY**

28) Do you or would you make a post-residency? * (mandatory, only one answer
 yes, as an assistant professor
 yes, as an hospital assistant specialist full time
 yes, as an hospital assistant specialist time-share between two or more hospitals
 yes, as a resident (supplemental year called “gold medal”)
 yes, as a contractual hospital practitioner
 yes, as attached practitioner
 no
 I do not know

29) Do you or did you have to wait six months of a year before starting a post-residency position? * (Mandatory, only one answer)
 yes
 no

30) Why do you realize or would you realize a post-residency? (Multiple answers possible)
 finish my training
 access to an university hospital career
 access to a career in public hospital or comprehensive cancer center
 access to “secteur 2” (a better remuneration in France)
 to validate a DESC
 other:

31) Would you or have you accepted a post residency as hospital assistant specialist timeshare between a university hospital or comprehensive cancer center and a non-teaching hospital)? (Only one answer)
 yes
 no

32) Why did not you accept or do not accept a post residency with timesharing exercise? (Multiple answers possible)
 inadequate pay
 inadequate remuneration of night duty
 transport times too large between the two workplace
 exercise not easily reconcilable speciality with a exercise at time-sharing

inadequate supervision to complete my training / improve myself
 no link with a university hospital or a comprehensive cancer center
 insufficient technical platform
 insufficient healthcare team
 inadequate medical activity
 exercise too general (by opposition to the hyperspecialization)

excessive workload
 difficulty in reconciling professional and personal life
 other:

33) Will you or have you changed your city for post-residency? (Only one answer)
 yes
 no
 I do not know

34) Why did you or will you plan to change your city to post-residency? (Multiple answers possible)
 to obtain a fellowship
 for reasons of geographical ties of fellowship area
 for reasons of family ties in the post-boarding area
 to follow my (my) partner (s)
 to access better quality training
 to access a university hospital career
 other:

**CAREER and PROFESSIONAL LIFE**

35) What career do you plan ? (mandatory, multiple answers possible)
 career in comprehensive cancer center without university function
 career in public hospital without university function
 career in public university hospital
 career in private hospital
 career mixed in the public and the private health care systems
 career in the pharmaceutical industry / manufacturer
 career with a research activity exclusively
 other:

36) Which working time would you wish to have? (only one answer)
 full-time
 70 to 90%
 half time
 other:

37) Are you worried for your future career? (Multiple answers possible)
 lack of job opportunities for myself
 lack of job opportunities for my partner in my exercise area
 excessive workload / schedule
 activity and / or type of exercise not in line with my professional goals
 poor quality of life
 insufficient incomes
 exercise of professional isolation (one team doctor)
 reconsideration of the freedom of installation
 other:

**RESEARCH**

38) Have you made a Master degree? * (Mandatory, only one answer)
 yes conditional go to question 39
 no: conditional go to question 50
 undecided: ongoing reflection / preparation: conditional go to question 50

39) How did you discipline your Master degree? (Pick one answer)
 pharmacology / therapeutic cancer
 cellular / molecular biology
 radiobiology
 medical physics
 medical imaging
 clinical research / methodology / biostatistics
 medical ethics
 oncogenetics
 bioinformatics
 other:

40) How optical fits this Master degree ? (Multiple answers possible)
 a university hospital career as a professor.
 an open mind, curiosity, attraction for research in general
 an additional training in clinical, basic or transfer research
 a pending a post-internship position
 a prerequisite for access to a post-internship position
 an obligation by the coordinator of des my university to validate my internship
 other:

41) What type of funding were you received for your Master degree? (Multiple answers possible)
 national funding ("research year")
 regional funding
 INSERM funding
 INCa funding
 The League against Cancer funding
 ARC Fundig
 scientific societies, foundations or related funding
 pharmaceutical industry / manufacturer funding
 self-financing: replacements, night duty, penalties, other ...
 no funding
 other:

42) Have you validated your Master degree? (Only one answer)
 yes
 no
 in progress

43) Your Master degree has been the subject of one or more scientific publications? (Multiple answers possible)
 yes (published or accepted)
 submitted
 no, refused
 no, not even considering the advancement of my research work

44) Your Master degree has there been one or more posters? (Multiple answers possible)
 yes (displayed or accepted)
 submitted
 no, refused
 no, not even considering the advancement of my research work

45) Your Master degree has there been one or more oral communications? (Multiple answers possible)
 yes, (presented or accepted)
 submitted
 no, refused
 no, not even considering the advancement of my research work

46) Are you satisfied with your Master degree?

0 1 2 3 4 5 6 7 8 9 10
select a range value between 0 = not at all satisfied and 10 = very satisfied

47) Are you satisfied with the supervision received for the realization of your diploma?

0 1 2 3 4 5 6 7 8 9 10
select a range value between 0 = not at all satisfied and 10 = very satisfied

48) Are you or are you going to extend your master by: (multiple responses) (only one answer)
 a PhD
 a mobility
 i do not intend to extend by a PhD or a mobility
 other:

49) Have you received funding for realize them? (Only one answer)

yes
 no
 in progress

50) Free comments
